# ApoER2 and VLDLr Are Required for Mediating Reelin Signalling Pathway for Normal Migration and Positioning of Mesencephalic Dopaminergic Neurons

**DOI:** 10.1371/journal.pone.0071091

**Published:** 2013-08-16

**Authors:** Ahmed Sharaf, Hans H. Bock, Björn Spittau, Elisabeth Bouché, Kerstin Krieglstein

**Affiliations:** 1 Institute for Anatomy and Cell Biology, Department of Molecular Embryology, Albert-Ludwigs-Universität, Freiburg, Germany; 2 Center for Neuroscience, Albert-Ludwigs-Universität, Freiburg, Germany; 3 Department of Medicine II, Albert-Ludwigs-Universität, Freiburg, Germany; University of Memphis, United States of America

## Abstract

The migration of mesencephalic dopaminergic (mDA) neurons from the subventricular zone to their final positions in the substantia nigra compacta (SNc), ventral tegmental area (VTA), and retrorubral field (RRF) is controlled by signalling from neurotrophic factors, cell adhesion molecules (CAMs) and extracellular matrix molecules (ECM). Reelin and the cytoplasmic adaptor protein Disabled-1 (Dab1) have been shown to play important roles in the migration and positioning of mDA neurons. Mice lacking Reelin and Dab1 both display phenotypes characterised by the failure of nigral mDA neurons to migrate properly. ApoER2 and VLDLr are receptors for Reelin signalling and are therefore part of the same signal transduction pathway as Dab1. Here we describe the roles of ApoER2 and VLDLr in the proper migration and positioning of mDA neurons in mice. Our results demonstrate that VLDLr- and ApoER2-mutant mice have both a reduction in and abnormal positioning of mDA neurons. This phenotype was more pronounced in VLDLr-mutant mice. Moreover, we provide evidence that ApoER2/VLDLr double-knockout mice show a phenotype comparable with the phenotypes observed for Reelin- and Dab1- mutant mice. Taken together, our results demonstrate that the Reelin receptors ApoER2 and VLDLr play essential roles in Reelin-mediated migration and positioning of mDA neurons.

## Introduction

Parkinson's disease (PD) is an age-associated neurodegenerative disorder characterized by the gradual degeneration and loss of dopaminergic neurons of the substantia nigra pars compacta (SNc) of the midbrain, which presents in the clinic as movement and behavioural disorders due to the reduction of dopamine levels in the striatum. Midbrain dopaminergic (mDA) neurons can be identified by the immunohistochemical detection of tyrosine hydroxylase (TH) [1]. Dopaminergic progenitor cells are generated in the mouse isthmic organizer located in the ventricular zone between E10 and E12 and migrate first ventrally along radial glial cells toward the pial surface of the midbrain and then laterally along tangential nerve fibers originating from the lateral part of the mesencephalon [2] to reach their final destinations. There they form three different neuronal populations, the substantia nigra pars compacta (SNc, A9), ventral tegmental area (VTA, A10) and retrorubral field (RRF) [3], [4], [5], [2] and [6]). Radial glial cell bodies are located in the vicinity of the ventricular zone and extend their processes throughout the whole ventral midbrain region until they reach the pial surface, thereby forming a scaffold [7]. The neurons migrating along the radial glia fibers are at different stages of maturation [6]. Midbrain dopaminergic neurons complete their migration at E18 in the mouse and start to extend their projections to the striatum between E16 and birth. However, many aspects of mDA neuronal migration remain to be elucidated [8]. Several molecular cues control the orientation and organization of mesencephalic dopaminergic neurons including neurotrophic factors, cell adhesion molecules (CAMs) and extracellular matrix molecules (ECM) [9]. Among these factors is Reelin, a critical regulator of neuronal migration in laminar brain structures including the cortex, hippocampus and cerebellum [10], [11], [12], [13], [14], [15], [16]. Reelin also regulates the migration and/or dendritic outgrowth of developing neurons [14], [17] through regulating cell adhesion and cytoskeletal dynamics. Reelin signals through binding to the lipoprotein receptors ApoER2 and VLDLr on responsive migrating neurons and/or radial glial cells, which transmit the Reelin signal via Src family kinases [18], [19], [20]. In reeler and yotari mice, which lack Reelin or Dab1 protein, respectively, there are fewer SNc dopaminergic neurons due to a defect in the proper migration and normal positioning, but not in the neurogenesis, of mDA neurons [7]. The migratory deficits in reeler mice might be due to cell autonomous effects and/or to the misregulated differentiation of radial glial cells, which have an important role in controlling neuronal migration [21]. Here, we report that both Reelin receptors (VLDLr and ApoER2) are required for the normal migration of mDA neurons. We demonstrate the impact of ApoER2 and VLDLr on the normal migration of mDA neurons using single ApoER2-, VLDLr- and ApoER2/VLDLr double- as well as Dab1-knockout mice. Our results indicate that the canonical Reelin signalling pathway mediated by ApoER2 and VLDLr is an essential contributor for the normal positioning of mDA neurons. We provide evidence that mDA neurons of ApoER2/VLDLr double knockout mice display a phenotype, which is similar to that of Reeler and Dab1-mutant mice.

## Materials and Methods

### Ethics Statement

The study was carried out in strict accordance with the National Health and ethical regulations and care of animals was in accordance with the institutional guidelines. The protocols used were approved by the Committee on the Ethics of Animal Experiments of the Albert-Ludwigs-University Freiburg (Permit number: X-11/18H). Animals were sacrificed under xylazin/ketamine anaesthesia and all efforts were made to minimize suffering.

### Animals

This study was carried out using several mutant mouse lines (Table 1). Male C57BL/6J littermates with wildtype, Dab1^−/−^, Apoer2^−/−^, VLDLr^−/−^ or Apoer2/VLDLr double-knockout (A/V dko) genetic backgrounds at different developmental stages (E18.5-P25) were used for this study. All animal experiments were carried out in accordance with the European Communities Council Directive and were further approved by the local government and chief veterinarian.

**Table 1:** Overview of mutant mice lines.

Genes	Mutation	References
**Dab1**	Exon 17, 18 replaced with pol2neo cassette	[22]
**ApoER2**	Amino acids 162–262 from exon 5 replaced with pol2neobpA cassette	[23]
**VLDLr**	Deletion of amino acids 27–69	[24]

Wildtype and homozygous Dab1- and VLDLr-mutant animals were obtained by the crossbreeding of heterozygous mice. In the case of the ApoER2-knockout mice, homozygous animals were generated by crossbreeding heterozygous parents or homozygous knockout females with heterozygous males, as homozygous males are infertile. The double Apoer2/VLDLr-knockout mice were all of a VLDLr^−/−^ genetic background [20]. The genotype of all animals was determined by PCR of tail DNA and confirmed for each animal used in this study. For timed pregnancies, the day of the vaginal plug was recorded as embryonic day 0.5 (E 0.5).

### Tissue preparation and embedding

Embryos were obtained by cesarean section at E18.5 and brains dissected and fixed by immersion in 4% paraformaldehyde (PFA). Postnatal mice were anesthetised using a ketamine 10% (20 mg/kg, Pfizer) and Rumpon 2% (4 mg/kg, Bayer Healthcare) mixture and transcardially perfused with 4% paraformaldehyde (PFA) in phosphate buffered saline (PBS; 0.9% NaCl, 0.01 M phosphate buffer). After perfusion, brains were dissected and post-fixed using 4% PFA in 0.1 M phosphate buffer overnight according to a standard protocol [25]. Subsequently, brains were incubated for 4 h in Bouin's solution and embedded in paraffin before sectioning with a microtome (Leica, Germany). Alternatively, brains were incubated in tissue freezing medium before sectioning with a cryostat (Leica, Germany). The embedded brains were cut into coronal serial sections of 10 µm thickness.

### Nissl-staining

Paraffin sections were deparaffinized, hydrated in distilled water and transfered to 0.5% cresyl violet solution for 7 min. The sections were rinsed in distilled water containing a few drops of glacial acetic acid, transferred to ascending grades of ethanol, and finally cleared with Rothihistol, before mounting using entellan (Merck, Germany).

### Immunohistochemistry

Sections were deparaffinized and rehydrated with a series of descending grades of ethanol, then washed with PBS for 3×5 min each. Antigen retrieval was carried out in 0.1 M citrate buffer solution (pH = 6) for 10 min using a microwave (600 Watt). Sections were washed for 3×5 min each and incubated with 10% hydrogen peroxide (H_2_O_2_) in methanol for 15 min to eliminate the endogenous peroxidase activity from the brain tissues. Sections were incubated with blocking solution (PBS containing 10% normal goat serum (NGS; life Technology, Germany) and 10% Triton-X-100 (Sigma, Germany)) to minimize non-specific labelling. Paraffin sections were incubated at 4°C overnight with primary antibodies (mouse monoclonal anti-TH, dilution 1∶2000, Millpore; rabbit polyclonal anti-calbindin, 1–1000, Chemicon; rabbit polyclonal anti-caspase, 1–300, Abcam) diluted in blocking solution (rabbit monoclonal anti-TH, dilution 1∶2000, Millipore) and then at room temperature for 1 hr in PBS containing goat anti-rabbit antibody conjugated to biotin (1∶300; Molecular probes). The sections were washed in PBS (3×5 min each) followed by incubation with an Avidin-Biotin complex (ABC, Vector Labs) for 1 hr. To visualize the staining, sections were incubated with a peroxidase substrate kit (SK 4700, Vector labs) for 10 min as described by Adams (1981). The sections were dehydrated with ascending grades of ethanol, cleared with Rothihistol and mounted using entellan (Merck, Germany).

### Double immunofluorescence (IF)

For immunofluorescence, the sections were treated as above except that for the secondary antibody labeling, sections were washed in PBS (3×5 min each) then incubated with donkey anti-rabbit antibody conjugated to Alexa Fluor® 568 (AF, 1∶500, Molecular Probe) or donkey anti-rabbit antibody conjugated to Alexa Fluor®488 (AF, 1∶500, Molecular Probe) for 1 hr in the dark at room temperature. Sections were then washed 3×5 min each in PBS and counterstained and mounted with Fluoromount-G containing 4′-6-diamidino-2-phenylindole (Dapi, SouthernBiotech) for nuclear staining. Sections were fixed into place using clear nail polish and visualized with a fluorescence microscope (Zeiss microscope, Göttingen, Germany).

### Unbiased stereological counting

Collected brains were fixed overnight in 4% PFA and cryoprotected through an ascending series of sucrose solutions (10% sucrose in PBS for 1 hr followed by 30% sucrose in PBS). The brains were embedded in tissue freezing medium (Leica, Germany) and 40 µm coronal sections were obtained throughout the midbrain including the entire SNc, and VTA of Apoer2^−/−^; Vldlr^−/−^; A/V dko and age-matched wildtype littermates (n = 3 per group). Afterwards, the sections were immunostained for TH as described above, with the exception that for secondary antibody labeling sections were incubated with goat anti-mouse IgG antibody conjugated to biotin (1∶300, Molecular probes) for 1 hr at room temperature. After washing for 3×5 min each with PBS, sections were incubated with Avidin-Biotin Complex (ABC, Vector Labs) for 2 hr at room temperature. The sections were then rinsed with PBS (3×5 min each) and stained with cresyl violet as a counter stain. Embedding was performed as described above. For a total estimate of TH-positive mesencephalic dopaminergic neuron numbers, we used the optical fractionator, an unbiased stereological method. Midbrain coronal serial sections including mesencephalic DA nuclei, VTA, SNc, and RRF were examined. The medial terminal nucleus (MTN) of the accessory optical tract was used as the medial border of the SNc [26] Sections used for counting covered the entire substantia nigra and VTA, starting with the first appearance of TH-positive neurons in the frontal midbrain and extending to the most caudal parts of the midbrain. This resulted in a total of six to eight sections sampled from the right hemisphere of each animal. Every third section was analyzed for cell counting in a rostral–caudal fashion using the Stereo-Investigator with optical fractionator according to the optical dissector rules [27] and adjusting the counting frame to (100×100) µm^2^. Cells with a darkly labeled cytoplasm and a clearly visible, unstained nucleus were included in the cell counts. The optical fractionator was optimized to reach a coefficient of error of ≤0.1. Cell counts were performed using a 63× oil objective (Zeiss, Göttingen, Germany) and AxioImager (Zeiss, Göttengen, Germany).

### Measurement of the optical density of striatal fibers

The striatal TH fiber density was assessed by optical density (OD) analysis. Images of stained striatal sections were captured using a Zeiss microscope (Zeiss, Göttingen, Germany). ODs were evaluated using ImagJ from striatal sections of at least three different animals, comparing the cortex facing the striatum. The result was calculated as an average of the ratio of striatal to cortical TH-staining from the same section.

### Statistics

Statistical analysis was performed using the software, GraphPad Prim 5 (GraphPad Software Inc.). For determination of the significant differences the unpaired t test was used. P-value lower than the significance level of 0.05 was considered statistically significant.

## Results

### ApoER2 and VLDLr are required for the normal positioning of mDA neurons

**Figure 1:**
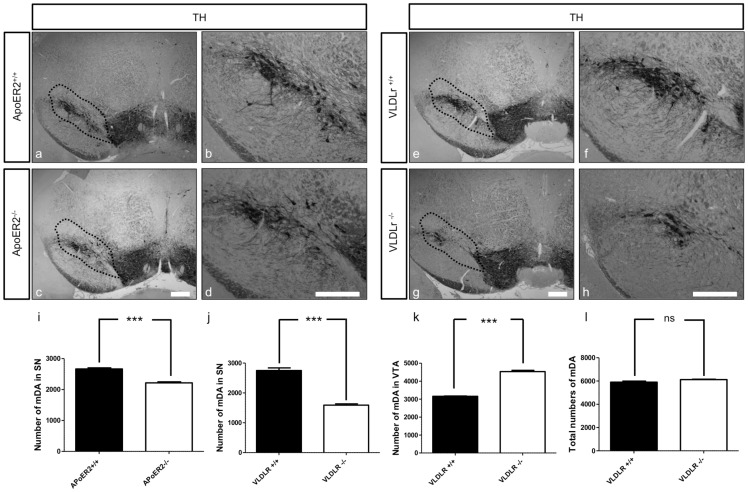
Reduction of nigral dopaminergic neurons in both ApoER2 and VLDLr mutant mice at P25. TH staining of mDA neurons of wildtype (a, e) and single ApoER2 (c) and VLDLr (g) mutant mice defined by dotted black line. (b, d, f, h) Higher magnification images for the areas defined by dotted black lines in (a, c, e, g) reveal a slight reduction of TH-positive neurons of SNc in ApoER2^−/−^ (d) and a pronounced reduction in VLDLr^−/−^ (h) as compared to corresponding wildtype littermate controls (b, f). Scale bars  = 200 µm. Stereological estimates of the total numbers of TH-positive neurons in SNc in ApoER2 animals (I), SNc, VTA and total numbers of mDA neurons (j, k and l respectively) in VLDLr animals, reveal about 15% and 40% reduction in ApoER2^−/−^ (i) and VLDLr^−/−^ (j) respectively, as compared to the wildtype animals, and significant increase of VTA neuron number in VLDLr^−/−^ (k). There was no significant reduction in the total numbers of mDA neurons between VLDLr^+/+^ and VLDLr^−/−^ (l). Three animals were used for each genotype for cell counting. Results were given as means ± SEM. P-values derived from unpaired t test are ***p<0.0007 in i; ***p<0.0003 in j; ***p<0.0001 in k.

Previously, it has been shown that Reelin has an important role for controlling the migration of nigral dopaminergic neurons [7]. To investigate the contribution and the impact of the Reelin receptors ApoER2 and VLDLr on the normal migration of mDA neurons in detail, we analyzed homozygous single ApoER2- and VLDLr-knockout mouse postnatal brains (P25-P30) by immunostaining for TH using serial coronal sections of whole midbrains of wildtype and mutant mice. We found a significant reduction in the total numbers of nigral dopaminergic neurons in ApoER2^−/−^ (Fig. 1c, d) and VLDLR^−/−^ mice (Fig. 1g, h) when compared to the corresponding wildtype littermates (Fig. 1a, b, e, f).The reduction of dopaminergic neurons in SNc was only apparent and significant in the intermediate sections of the midbrain. The abnormal positioning and reduction of TH-positive neurons in the SNc was stronger and more pronounced in VLDLr^−/−^ than in ApoER2^−/−^ mice. By contrast, the rostral sections of the SNc in both ApoER2- and VLDLr-single mutant mice were normal when compared to the wildtype animals (data not shown). Stereological counting of TH-positive neurons in the SNc revealed a ∼15% reduction in ApoER2^−/−^ (Fig. 1i) and ∼40% reduction in VLDLr^−/−^ mice (Fig. 1j). However, there was an increase in the mean total number of TH-positive dopaminergic neurons in the VTA of VLDLr-mutant mice when compared to wildtype mice (Fig. 1k), although the total estimated number of mDA neurons was not significantly different (Fig. 1l), suggesting that the disorganization and abnormal positioning of midbrain dopaminergic neurons in the absence of ApoER2 or VLDLr might be related to a failure of the nigral neurons to reach their proper location in the lateral mesencephalon. These data indicate that the Reelin receptors ApoER2 and VLDLr contribute to the normal lateral and tangential migration of mDA neurons.

### ApoER2/VLDLr double-knockout mice have the same phenotype as Dab1 mutant mice

**Figure 2:**
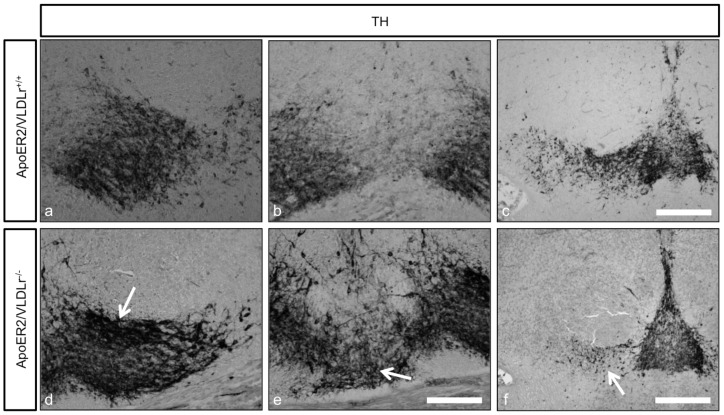
TH immunostaining of representative sections from mDA neurons of E18.5 mice. Normal differentiation of mDA into SNc and VTA (a, b) and normal distribution of RRF neurons (c) in wildtype. In A/V dko there was nigral neuron loss and accumulation of TH cells in VTA (d, e, arrow) in addition to the alteration in distribution and reduction in numbers of RRF neurons (f, arrow). Scale bars 100 µm in a, b, c, d and 200 µm in c, f.

**Figure 3:**
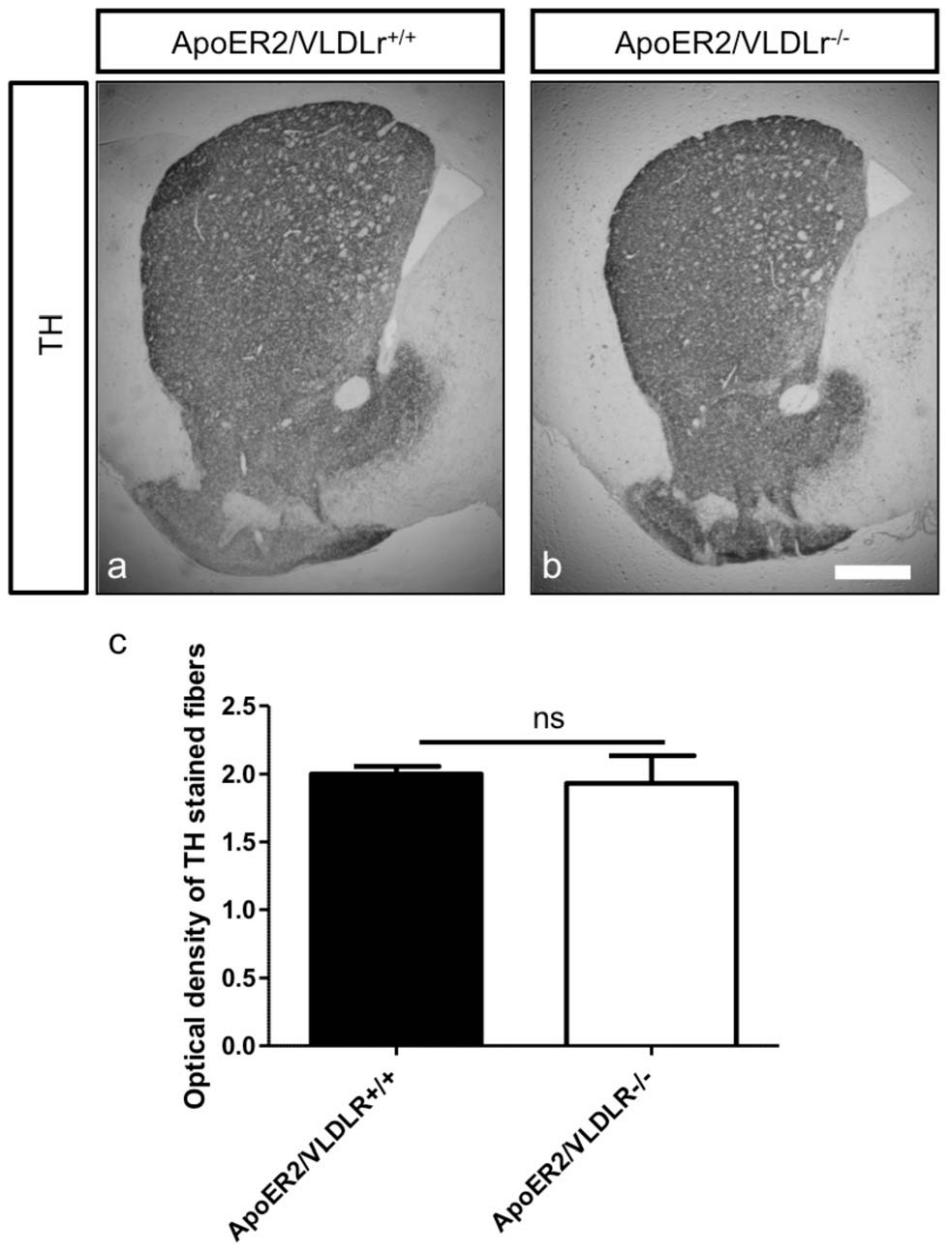
Loss of nigral dopaminergic neurons in A/V dko mice at P15. TH immunostaining of representative sections from mDA neurons of wildtype (a, b) and A/V dko (c, d). The rostral and intermediate sections of SNc in wildtype reveal correct positioning and normal migration of nigral neurons. However, in A/V dko the nigral neurons in the rostral sections appear irregularly organized (c), in addition to a pronounced loss of SNc neurons, which appear located lateral to VTA neurons (d). (e, f) Stereological counting of the total numbers of TH- positive neurons in SNc and VTA respectively reveals a significant reduction in the number of nigral neurons of A/V dko as compared to wildtype (e). However, in (f) a pronounced increase in VTA neuron numbers of A/V dko is seen. Three animals were used for each genotype for cell counting. Results are given as mean ± SEM. P-value derived from unpaired t-test are ***p<0.0001. Scale bar  = 200 µm.

**Figure 4:**
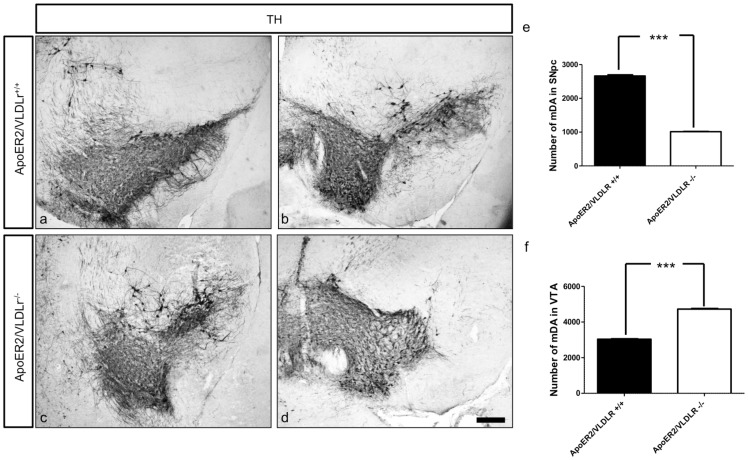
Analysis of striatal TH fibre density at P15. TH staining of striatal sections of wildtype (a) and A/V dko (b). The TH staining of striatum indicates normal projections of SNc neurons despite their aberrant positioning in the VTA in A/V dko. Scale bar  = 500 µm. (c) Measuring the optical density of TH-positive fibres reveals no significant difference between wildtype and mutant mice.

**Figure 5:**
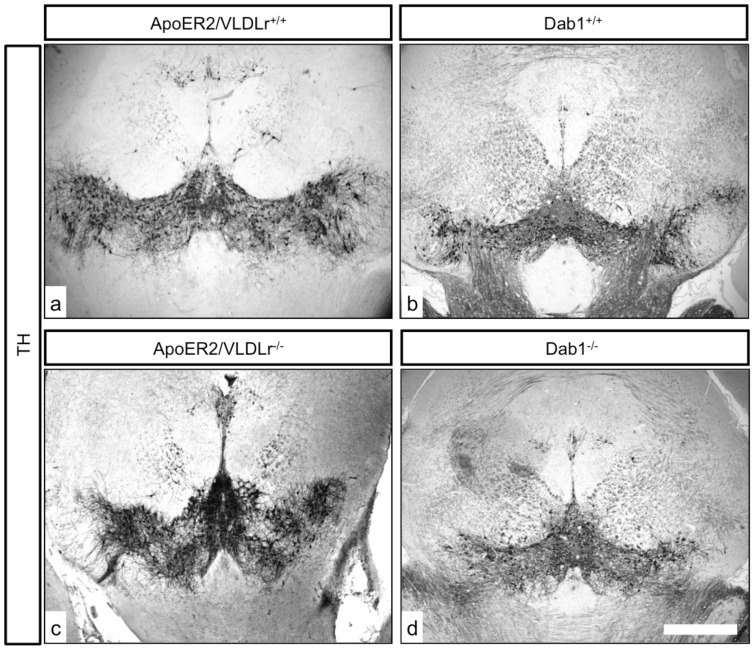
Analysis of RRF of mDA neurons at P15. TH staining of representative RRF coronal sections of wildtype (a, b), A/V dko (c) and Dab1^−/−^ (d) mice. (a, b) show the correct positioning and normal distribution of RRF in the wildtype. However, (c, d) reveal a severe alteration in the distribution of dopaminergic neurons of RRF and a reduction in numbers in A/V dko and Dab1−/−. Scale bar  = 500 µm.

In addition to the effect of single deletion of ApoER2 or VLDLr on the normal migration of mDA we also studied the phenotype of mDA neurons in ApoER2/VLDLr double-knockout mice (A/V dko), which we found was similar to Reeler and Dab1-deficient mice [7]. This phenotype was detected at E18.5 of A/V dko (Fig. 2d, e, compared to wildtype, Fig. 2a, b). In addition, we found a disorganization of the RRF in A/V dko (Fig. 2f) as compared to wildtype mice. This phenotype was also seen in the postnatal brain. The nigral dopaminergic neurons showed a disorganized and irregular distribution in the rostral sections of SNc (Fig. 3c) and a great reduction in the intermediate sections of A/V dko (Fig. 3d). A 54% reduction in the number of SNc neurons was seen by stereological counting of TH-positive neurons in A/V dko when compared to wildtype (Fig. 3e). However, there was a significant increase in the number of VTA neurons of A/V dko when compared to wildtype animals (Fig. 3f). By measuring the optical density of TH-stained fibers (Fig. 4a, b), we found no significant difference in the striatum in both wildtype and A/V dko animals (Fig. 4c). The abnormal positioning of mDA neurons in A/V dko mice did not only affect SNc neurons but also the RRF, which shows an alteration, abnormal distribution (Fig. 5C) and reduction in the number when compared to wildtype (Fig. 5a, b). These results demonstrate that A/V dko mice have a similar midbrain phenotype to Dab1-mutant mice (Fig. 5e). Finally, we concluded that the rostral sections of nigral dopaminergic neurons were only slightly affected in A/V dko animals. However, the nigral neurons in the middle sections failed to reach their final destination in the lateral mesencephalon and were aberrantly located lateral to the VTA in A/V dko animals. In addition, we found the distribution of RRF neurons was altered.

### The mDA neuronal phenotype in Dab1-mutant mice in comparison to Reelin receptor-mutant mice

**Figure 6:**
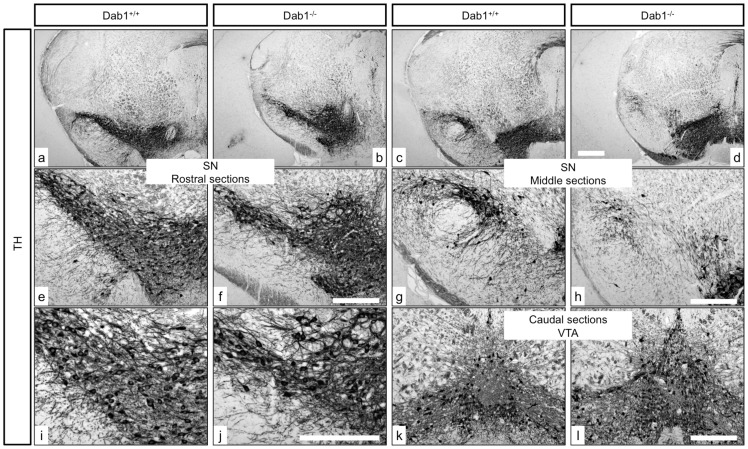
Analysis of mDA neurons in Dab1−/− mice. TH immunostaining of representative sections of rostral sections of SN in wildtype (a) and Dab1 knockout littermates (b). (e, i) Higher magnification images reveal correct positioning, normal distribution and morphology of dopaminergic neurons in the rostral sections of wildtype mice. (f, j) show slight defect in the migration of the rostral sections of SNc neurons in Dab1 mutant mice. TH immunostaining of mDA neurons in intermediate sections in both wildtype (c) and Dab1^−/−^ mice (d). (g, h) Higher magnification images reveal significant reduction of nigral dopaminergic neurons in Dab1^−/−^ (h) as compare to wildtype (g). TH immunostaining of the caudal mDA neurons showing higher numbers of TH positive neurons in VTA of Dab1^−/−^ (k) as compare to Dab1^+/+^ (m). Scale bars = 200 µm.

**Figure 7:**
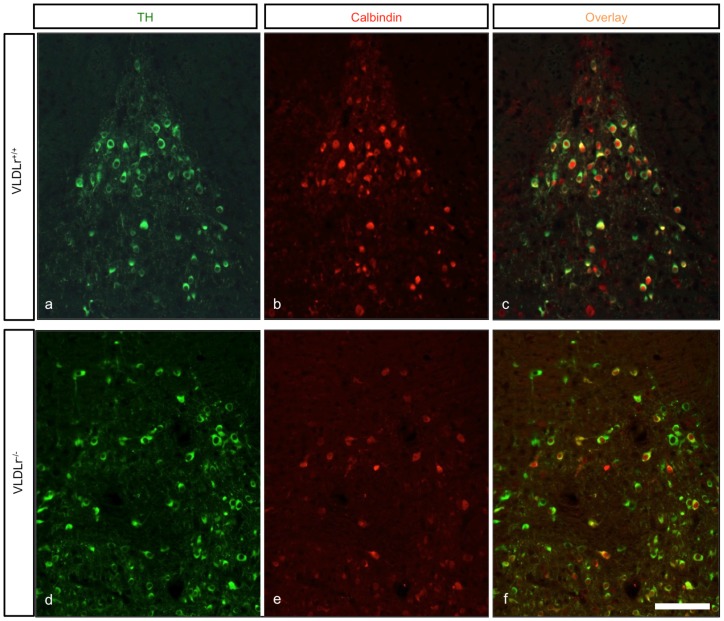
Analysis of the caudal sections of mDA neurons. (a–f) Coronal brain sections stained with TH (green) and calbindin (red) of VLDLr^+/+^ and VLDLR^−/−^ mice. (a–c) in VLDLr^+/+^ mice, all TH positive neurons of VTA coexpress calbindin. In VLDLr^−/−^ (d–f) there were more TH positive cells in VTA than in VLDLr^+/+^ VTA and not all TH neurons coexpress calbindin. Scale bar = 100 µm.

**Figure 8:**
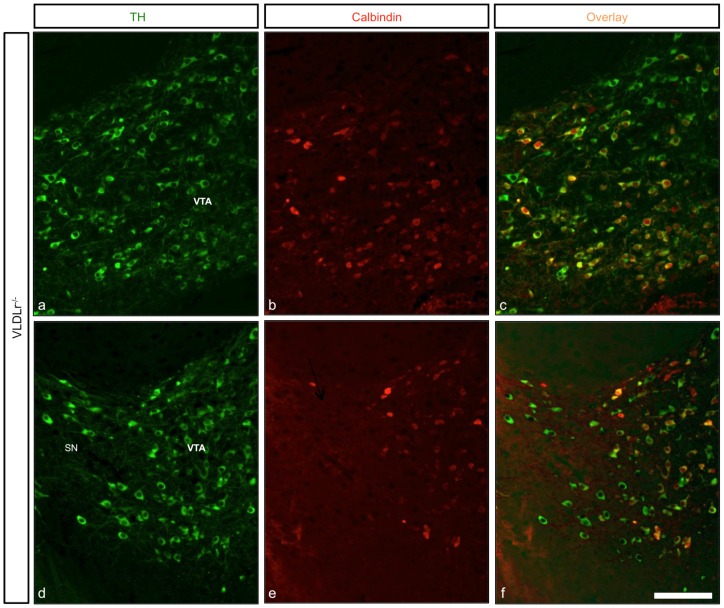
Analysis of the intermediate sections of mDA neurons in VLDLr^−/−^. (a–f) Midbrain coronal sections stained with TH (green) and calbindin (red). (a, d) TH staining of VTA neurons (a) and the line of demarcation between VTA and SNc. (b, e) Calbindin immunostaining of mDA neurons of VLDLr^−/−^ mice. (c, f) Merge of images in a, b and d, e. Not all VTA neurons (c) and also nigral neurons (f) were positive for calbindin. Scale bar  = 100 µm.

By analysing nigral neurons in wildtype (Fig. 6a) and Dab1^−/−^ mice (Fig. 6b), we observed an irregular distribution of TH-positive neurons in the rostral sections of the SNc of Dab1^−/−^ (Fig. 6f, j) as compared to wildtype littermates (Fig. 6e, i). Moreover, there was a great reduction of TH-positive neurons in the intermediate sections of the SNc in Dab1^−/−^ animals (Fig. 6d, h). In addition, more TH-ir neurons were located in the caudal sections of the VTA in Dab1^−/−^ animals (Fig. 6l). Next, we immunostained coronal brain sections from ApoER2-, VLDLr-, A/V dko- and Dab1-mutant animals with calbindin, a specific marker for the VTA dopaminergic neurons that does not label nigral neurons, to confim that the reduction of SNc dopaminergic neurons was due to a defect in the normal migration of mDA neurons. In VLDLr-mutant mice, not all TH-positive dopaminergic neurons in the VTA colocalized with calbindin (Fig. 7c). TH-positive cells accumulated lateral to VTA neurons (Fig. 7c). However, in age-matched wildtype littermates allmost TH-positive neurons in the VTA also colocalize with calbindin (Fig. 7d–e). In addition, we found more TH-positive neurons in the VTA of mutant VLDLr animals (Fig. 7d, f) when compared to the VTA of wildtype animals (Fig. 7a–c). Moreover, Fig. 8a–c shows that in VLDLr-mutant mice, many TH-positive neurons did not express calbindin in the intermediate sections of the VTA. Fig. 8d shows the line of demarcation between SNc and VTA neurons. Moreover, the ectopic nigral neurons were located in the lateral VTA of VLDLr^−/−^ mice (Fig. 8). Counting of TH/calbindin double-positive neurons in the VTAs of wiltype and VLDLr mutant mice revealed that in the wildtype 90,91% (±3,078%) of all TH-positive neurons were also calbindin-positive. However, in the VLDLr mutants only 59,08% (±0,973%) of the TH-positive neurons were also calbindin-positive. These results indicate that the neurons with the destitaion of the SNc are mislocated in the VTA of VLDLr mutant mice.

### The reduction in dopaminergic neuron numbers in the SNc results from a migration defect rather than from increased cell death

**Figure 9:**
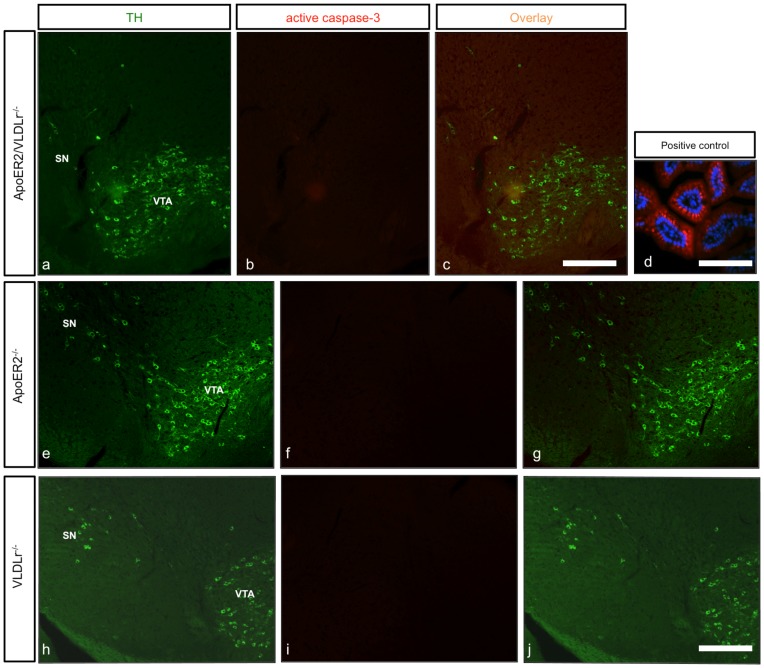
Detection of active caspase-3 in the mesencephalic dopaminergic neurons. (a–j) Coronal paraffin brain sections of ApoER2/VLDLr^−/−^ (a–c), ApoER2^−/−^ (e–g) and VLDLr^−/−^ mice (h–J), stained with anti-active caspase-3 antibody (red) and TH (green). In all mutant mice (b, f, i) no positive signals for active caspase-3 were detected in SNc (a, e, h). (d) Positive control for active caspase-3: cells of intestinal villi, nuclei counterstained with Dapi (blue). (c, g, j) Merge of images in a, b; e, f, and h, i respectively. SN: Substania nigra, VTA: Ventral tegmental area. Scale bars = 200 µm.

The normal positioning and migration of mesencephalic dopaminergic neurons requires both ApoER2 and VLDLr. The localization of dopaminergic neurons in the mesencephalon of ApoER2-, VLDLr- and Dab1-mutant mice appeared normal. We sought to validate our results by excluding other possible reasons for a reduction of nigral dopaminergic neurons. Hence we performed immunostaining of coronal midbrain sections of single ApoER2-, VLDLr- and ApoER2/VLDLr double -mutant mice with an active caspase-3 antibody. We found that apoptosis (PCD) was not detected in ApoER2/VLDLr^−/−^ (Fig. 9a–c), ApoER2^−/−^ (Fig. 9e–g) and VLDLr^−/−^ mice (Fig. 9h–j), suggesting that the significant reduction of nigral neurons was not due to cell death.

## Discussion

Reelin is a key regulator of brain development during embryogenesis and also during postnatal life, regulating normal migration, differentiation and synaptic circuit formation [11], [12], [13], [14], [28]. Because of the previously described role of Reelin and its mediator, Dab1, on midbrain development [7]. We studied the effect of Reelin receptor (ApoER2 and VLDLr) deletion on normal mDA neuronal development during embryonic and postnatal stages.

### Both ApoER2- and VLDLr-mediated Reelin signaling are involved in the migration of mDA neurons

We studied the effect of the Reelin receptors ApoER2 and VLDLr on normal mDA neuronal development during embryonic and postnatal stages using receptor-deficient mice. The mDA neurons expressing TH first appear in the neuroepithelium at E10 and then migrate in three steps. [6] described the normal migration of mDA neurons until they reach their final location in the lateral mesencephalon. First, postmitotic mesencephalic neurons migrate radially along radial glial fibers until they reach the ventromedial mesencephalon located along the ventral pial surface at E14. Second, the dopaminergic neurons migrate laterally and completely differentiate into VTA and SNc mDA neurons. Our results show that both ApoER2 and VLDLr exert different effects during the final step of mesencephalic dopaminergic neuron migration, the lateral migration step.

We also observed in our work that the nigrostriatal system expressed all the Reelin signaling pathway components, including VLDLr, ApoER2 and Dab1, at the time when nigral dopaminergic neurons proceed to migrate laterally to locate in the lateral mesencephalon. However, Reelin was only located around the perikarya of dopaminergic neurons but not within the cells and was secreted from striatal patch neurons at E15 [29]. VLDLr-knockout mice display a more pronounced abnormal positioning of mDA neurons and a robust reduction of substantia nigra neuron numbers when compared to ApoER2-deficient mice. In addition to the reduction in numbers, VLDLr-knockout mice display alterations in the distribution of dopaminergic neurons in the RRF and an accumulation of nigral neurons in the VTA of the mesencephalon due to abnormal migration.

In E14 ApoER2-, VLDLr- and also ApoER2/VLDLr double-knockout mice, the mDA neurons were located normally in the ventral mesencephalon near the pial surface. Which are similar to the radial glia cells, appeared normally arranged and without any morphological changes in reeler mice [7]. This suggests that Reelin signaling mediated by ApoER2 and VLDLr receptors is only needed during the lateral migration step of mDA neurons, but not during their initial radial migration. Deletion of Reelin signaling pathway components caused different migratory defects depending on which signaling component was knocked out. It has been shown that cortical and hippocampal neurons in ApoER2-mutant mice suffer from migratory defects that are more pronounced when compared to VLDLr-mutant mice. However, the cerebellar defects were more exaggerated in VLDLr^−/−^ animals when compared to ApoER2^−/−^ animals [30], [23]. In A/V dko mice, we observed effects on normal positioning and migration of substantia nigra neurons that were comparable to reeler and Dab1-mutant mice [7]. This indicates that both receptors are important for mediating Reelin signaling in the midbrain.

During brain development, there are a series of migratory waves in which postmitotic precursor cells travel from the place of origin in the subventricular zone and migrate along radial glial cells to reach their target place through radial migration [31], [32], [33]. Kang and his colleagues suggested that the abnormal positioning of the mDA neurons was due to a poor arrangement of tangential nerve fibers in Reeler and Yotari mice [7]. It is worth to discuss that the initial formation of radial glia fibres is dramatically impaired after genetic ablation of Reelin receptors in mice and, thus, mDA neurons are no longer able to properly migrate from the medial midbrain structures to the lateral parts that later in development become the substantia nigra. In this scenario the misguided radial glia formation is the primary effects and the migratory defects of mDA neuron is secondary phenomenon.

In the current work we found a similar phenotype of migration defects in mDA neurons of A/V dko mice. We hypothesize that ApoER2 and VLDLr participate in the normal positioning of nigral dopaminergic neurons by controlling lateral migration of mDA neurons, either through regulating the normal detachment of dopaminergic neurons from the radial glial cells after they reach the ventral midbrain or by instructing the migrating neurons to complete their final step to reach the proper location in the lateral mesencephalon.

### mDA neuronal fibre projection

Measuring the striatal density of TH-positive fibres in all mutant genotypes showed no significant difference to age-matched wildtype littermates. This might indicate that either the SNc neurons normally project to the dorsal striatum, despite their aberrant position in the VTA and similar to the findings of [29] or the nigral dopaminergic neurons which succeeded in reaching their proper position projecting to the dorsal striatum and compensate for the processes of misplaced neurons.

### Migratory defects but not cell death

We confirmed that the loss of dopaminergic neurons in the SNc was mainly because of migration defects but not due to defects in the generation or the survival of mDA neurons. Our interpretation is based on three pieces of evidence.

First, the estimated total numbers of mDA neurons were not significantly different in single ApoER2-, VLDLr- and ApoER2/VLDLr double-mutant animals in comparison to wildtype littermates. These findings are contrary to the observations of Won and Zhao, who found a significant increase in the numbers of astrocytes and decrease in the numbers of newly generated neurons in adult reeler mice [34], [35]. Second, using calbindin as a marker for VTA neurons, but not SNc neurons, all TH-positive dopaminergic neurons in the VTA coexpressed calbindin in wildtype animals. However, in mutant animals there were many TH-positive cells in the VTA that did not colocalize with calbindin. This indicates that the nigral neurons that fail to migrate stay close to the VTA neurons.

Finally, we confirmed that there were no signs of apoptotic cell death in the SNc in all mutant animal lines (ApoER2-, VLDLr-, A/V dko- and Dab1-mutant mice) by using active caspase-3 staining. Our results regarding the abnormal positioning and defects in the migration of mesencephalic dopaminergic neurons in A/V dko mice were similar to findings in laminated brain structures (especially the neocortex, hippocampus and cerebellum) [23], [36], [37]. The same phenotype also extends to include the migration of sympathatic preganglionic neurons in the spinal cord. However, our result is contrary to the findings of [38] in the migration of hindbrain motor neurons in A/V dko mice. They found that the migratory phenotype of hindbrain motor neurons was the same in Reeler and Dab1-mutant mice but not in A/V dko mice, indicating that other Reelin receptors might be involved.

## Conclusion

Reelin is a key regulator of normal neuronal migration in many structures of the central nervous system, its effects being mediated by the Reelin receptors ApoER2 and VLDLr. We found that both ApoER2 and VLDLr are also required in mediating the effects of Reelin on the correct positioning and migration of nigral dopaminergic neurons and dopaminergic neurons of the RRF.
